# Efficacy and outcome of expanded newborn screening for metabolic diseases - Report of 10 years from South-West Germany *

**DOI:** 10.1186/1750-1172-6-44

**Published:** 2011-06-20

**Authors:** Martin Lindner, Gwendolyn Gramer, Gisela Haege, Junmin Fang-Hoffmann, Karl O Schwab, Uta Tacke, Friedrich K Trefz, Eugen Mengel, Udo Wendel, Michael Leichsenring, Peter Burgard, Georg F Hoffmann

**Affiliations:** 1 Centre for Paediatric and Adolescent Medicine, University Heidelberg, Heidelberg, Germany; 2 Centre for Paediatric and Adolescent Medicine, University Freiburg, Freiburg, Germany; 3 Children's Hospital, Klinikum am Steinenberg, Reutlingen, Reutlingen, Germany; 4 Centre for Paediatric and Adolescent Medicine, University Mainz, Mainz, Germany; 5 Centre for Paediatric and Adolescent Medicine, University Düsseldorf, Düsseldorf, Germany; 6 Centre for Paediatric and Adolescent Medicine, University Ulm, Ulm, Germany

**Keywords:** newborn screening, tandem-mass spectrometry

## Abstract

**Background:**

National newborn screening programmes based on tandem-mass spectrometry (MS/MS) and other newborn screening (NBS) technologies show a substantial variation in number and types of disorders included in the screening panel. Once established, these methods offer the opportunity to extend newborn screening panels without significant investment and cost. However, systematic evaluations of newborn screening programmes are rare, most often only describing parts of the whole process from taking blood samples to long-term evaluation of outcome.

**Methods:**

In a prospective single screening centre observational study 373 cases with confirmed diagnosis of a metabolic disorder from a total cohort of 1,084,195 neonates screened in one newborn screening laboratory between January 1, 1999, and June 30, 2009 and subsequently treated and monitored in five specialised centres for inborn errors of metabolism were examined. Process times for taking screening samples, obtaining results, initiating diagnostic confirmation and starting treatment as well as the outcome variables metabolic decompensations, clinical status, and intellectual development at a mean age of 3.3 years were evaluated.

**Results:**

Optimal outcome is achieved especially for the large subgroup of patients with medium-chain acyl-CoA dehydrogenase deficiency. Kaplan-Meier-analysis revealed disorder related patterns of decompensation. Urea cycle disorders, organic acid disorders, and amino acid disorders show an early high and continuous risk, medium-chain acyl-CoA dehydrogenase deficiency a continuous but much lower risk for decompensation, other fatty acid oxidation disorders an intermediate risk increasing towards the end of the first year. Clinical symptoms seem inevitable in a small subgroup of patients with very early disease onset. Later decompensation can not be completely prevented despite pre-symptomatic start of treatment. Metabolic decompensation does not necessarily result in impairment of intellectual development, but there is a definite association between the two.

**Conclusions:**

Physical and cognitive outcome in patients with presymptomatic diagnosis of metabolic disorders included in the current German screening panel is equally good as in phenylketonuria, used as a gold standard for NBS. Extended NBS entails many different interrelated variables which need to be carefully evaluated and optimized. More reports from different parts of the world are needed to allow a comprehensive assessment of the likely benefits, harms and costs in different populations.

## Introduction

The advent of tandem mass spectrometry (MS/MS) allowed for a substantial increase in the number of disorders included in the newborn screening (NBS) panel [1]. At present national NBS programmes differ widely. The American College of Clinical Genetics proposed 29 core and 25 secondary conditions [2], the German panel includes 12 metabolic disorders [3], the United Kingdom (UK) screens for phenylketonuria and medium-chain acyl-CoA dehydrogenase deficiency [4], France for phenylketonuria only [5], and Hongkong for no metabolic disorder but hypothyroidism [6]. Recommendations for the screening process also vary, e.g. for time of blood sampling between 24 hours (USA), 48 to 72 hours (Germany) to 120 hours (UK). Laboratory cut-offs and algorithms for confirmatory diagnostics are also not standardised.

Although the criteria proposed by Wilson and Jungner in 1968 [7] for NBS programmes are still accepted [2], they have been modified mainly driven by new technologies but without systematic evaluation of treatment and outcome yet [8]. Programme effectiveness, quality assurance and programme evaluation have been suggested as amendments to the Wilson and Jungner criteria [9,10].

## Patients and methods

### Panel of screened disorders

During a pilot period from January 1999 until April 2005 the panel of disorders was not officially regulated in Germany, and all disorders recommended in the US panel were screened for in our centre (N = 583,553 neonates). In December 2004 the regulatory authority for NBS was transferred to a national commission resulting in an officially implemented panel of 12 defined metabolic disorders to be exclusively screened from May 2005 onwards (N = 500,642 neonates) (Table 1 superscript a)[3]. At the same time written informed consent became mandatory. The recommended time for blood sampling was between day of life three to five before 2002 and between 36 and 72 hours thereafter [11].

**Table 1:** Patient sample, diagnostic criteria and treatment modalities

Disorder	N	Group 1 NBS	Group 2 symptomatic	Group 3 high risk	Treatment			Confirmation of diagnosis Minimal criteria to accept diagnosis as confirmed
					
					Yes	No	Un-known	
**Amino acid disorders/ Urea cycle disorders**								

PKU^a,f^	85	84		1^b^	79	0	6	Blood phenylalanine ≥ 600 μmol/L, normal urinary pterins and DBS dihydropteridine reductase activity

MHP^a,f^	88	88			2	61	25	First and control phenylalanine levels < 600 μmol/L, phenylalanine/tyrosine < 3, normal urinary pterins and DBS dihydropteridine reductase activity

PTPSD^e^	1	1			1			Characteristic pterin profile in urine

MSUD^a, e^	7	4	1	2	7			Elevated branched-chain amino acids including alloisoleucine

TYR I^e^	2	2			2			Elevated succinylacetone in urine

TYR III^f^	1	1			1			Tyrosine persistently > 500 μM, succinylacetone in urine normal, no clinical indication for tyrosinaemia type II or liver dysfunction

Homocystinuria^f^	0							Characteristic profile of homocysteine, methionine, cysteine in plasma

NKH^c,f^	1	1				1		Elevated glycine in plasma and CSF and pathological ratio plasma/CSF glycine

CIT I classic^e^	4	1	3		3		1	Characteristic amino acid profile in plasma and ammonia in blood

CIT I mild^f^	6	6			1	5		Characteristic amino acid profile in plasma and ammonia in blood, molecular genetic analysis

ASLD^e^	1			1	1			Characteristic amino acid profile in plasma and urine

Arginase deficiency^f^	0							Enzyme deficiency in erythrocytes

**Fatty acid oxidation disorders**								

CPT ID^a,e^	1	1			1			Enzyme deficiency in fibroblasts

CPT IID^a,e^	1	1			1			Enzyme deficiency in fibroblasts

CACTD^a,e^	0							Enzyme deficiency in fibroblasts

CTD^e^	3	3			3			Pathological tubular carnitine reabsorption, fibroblast transport studies

MCADD^a,e^	81	77	2	2	70	1	10	Characteristic acylcarnitine profile in plasma/DBS and/or hexanoylglycine in urine and/or informative genotype

LCHADD/ mTFP^a,c,e^	6	5		1	5		1	Characteristic acylcarnitine profile in plasma/DBS and/or informative genotype and/or enzyme activity

VLCADD^a,e^	6	6			6			Characteristic acylcarnitine profile in plasma/DBS and/or enzyme activity in lymphocytes or fibroblasts and/or informative genotype

SCADD^f^	9	9				3	6	Characteristic acylcarnitine profile in plasma/DBS and ethylmalonic acid in urine and enzyme activity

MADD^e^	3	3			2		1	Characteristic profiles of acylcarnitines in plasma and organic acids in urine

**Organic acid disorders**								

GA I^a,e^	6	6			6			Characteristic urinary organic acid profile, informative genotype

IVA classic^a,e^	5	4	1		5			Characteristic urinary organic acid profile

IVA mild^a,f^	10	9		1	10			Characteristic urinary organic acid profile, informative genotype

MBD^f^	0							Characteristic urinary organic acid profile

MMA/Cbl^e^	4	3	1		4			Characteristic urinary organic acid profile +/- abnormal concentrations of plasma homocysteine and methionine (complementation studies in fibroblasts in all patients)

PA^e^	4	3	1		4			Characteristic urinary organic acid profile

3-MCCD^f^	8	8			5	2	1	Characteristic urinary organic acid profile

MHBD^e^	0							Characteristic urinary organic acid profile, informative genotype

MGA^e^	0							Characteristic urinary organic acid profile, informative genotype

HCSD^e^	0							Characteristic urinary organic acid profile, enzyme deficiency

BIOD^a,e^	9	9			7		2	Enzyme deficiency in DBS and/or serum

KTD^e^	0							Characteristic profiles of acylcarnitines in plasma and organic acids in urine

HMG-CoA LD^e^	1	1			1			Characteristic profiles of acylcarnitines in plasma and organic acids in urine

**Other disorders**								

Galactosaemia^a,e^	14	9	2	3	12		2	Enzyme deficiency and elevated galactose-1-phosphate in DBS and erythrocytes

**Others conditions**								

Maternal 3-MCCD^f^	6	6				6		Characteristic acylcarnitine profile in plasma/DBS of mother and clearing of the pathological profile in the newborn

Not confirmed^d^	4	4				3	1	

**Total**	377	355	11	11	239	82	56	

^a ^Legal screening panel in Germany from April 1^st ^2005 onward

^b ^Diagnosed by prenatal diagnosis

^c ^Deceased: One child with mTFP at 6 months, one child with NKH in the neonatal period

^d ^Screening diagnoses: 1 CTD lost to follow up; 1 MMA lost to follow up; 1 MCADD, gestational age = 26 wks., deceased in neonatal period; 1 TYR I, gestational age = 25 wks., deceased in neonatal period

^e ^Disorder with risk of metabolic decompensation

^f ^Disorder without risk of metabolic decompensation

Abbreviations: ASLD = Argininosuccinate lyase deficiency; BIOD = Biotinidase deficiency; CACTD = Carnitine acylcarnitine translocase deficiency; CIT I = Citrullinaemia type I; CPT ID = Carnitine palmitoyltransferase I deficiency; CPT IID = Carnitine palmitoyltransferase II deficiency; CTD = Carnitine transporter deficiency; GA I = Glutaric aciduria type I; HCSD = Holocarboxylase synthetase deficiency; HMG-CoA LD = 3-Hydroxy-3-methylglutaryl-CoA lyase deficiency; IVA = Isovaleric aciduria; KTD = β-Ketothiolase deficiency; LCHADD = Long-chain 3-hydroxy-acyl-CoA dehydrogenase deficiency; mTFP = mitochondrial tri-functional protein deficiency; MADD = Multiple acyl-CoA dehydrogenase deficiency; MCADD = Medium-chain acyl-CoA dehydrogenase deficiency; MBD = 2-Methylbutyryl-CoA dehydrogenase deficiency; 3-MCCD = 3-Methylcrotonyl-CoA carboxylase deficiency; MGA = 3-Methylglutaconic aciduria; MHBD = 2-Methyl-3-hydroxybutyryl-CoA dehydrogenase deficiency; MHP = Mild hyperphenylalaninaemia; MMA/Cbl = Methylmalonic acidurias (all kinds); MSUD = Maple syrup urine disease; NKH = Non-ketotic hyperglycinaemia; PA = Propionic aciduria; PKU = Phenylketonuria; PTPSD = 6-Pyruvoyltetrahydropterin synthase deficiency; SCADD = Short-chain acyl-CoA dehydrogenase deficiency; TYR I/III = Tyrosinaemia type I/III; VLCADD = Very long-chain acyl-CoA dehydrogenase deficiency

### Population

Between January 1, 1999 and June 30, 2009, the NBS centre of the University of Heidelberg analysed dried blood spots of 1,084,195 neonates from three South-Western German states. Ninety percent of NBS samples were sent from obstetric units or children's hospitals and 10% from midwives or general paediatricians. MS/MS NBS was performed as described previously in a preliminary report from our centre [12].

### Cases with confirmed diagnosis of a metabolic disorder

In 377 cases confirmatory diagnostics was recommended. A metabolic disorder was confirmed in 373 cases. Minimal criteria for accepting a diagnosis as confirmed are stated in Table 1. In four cases a disorder was suspected, but further confirmatory investigation was not possible due to early death (one suspicion of tyrosinaemia type I, one of medium-chain acyl-CoA dehydrogenase deficiency) or because cases were lost to follow-up (one suspicion of methylmalonic aciduria, one of carnitine transporter deficiency) (Table 1). Maternal 3-methylcrotonyl-CoA carboxylase deficiency was diagnosed in 6 of the 373 cases. To our knowledge there has been no false negative screening result. Positive screens were communicated by phone as well as by fax and/or mail for all samples but for hyperphenylalaninaemias, where a second sample was only requested by fax and/or mail. The study sample was subdivided into three groups: Group 1 (NBS) comprised 355 neonates with a high suspicion of a metabolic disorder resulting from regular NBS, group 2 (symptomatic) contained 11 patients diagnosed because of clinical symptoms before NBS blood sample was taken or before NBS result was available. In group 3 (high risk, 11 patients) specific metabolic analyses were performed immediately after birth (n = 10) or even prenatally (n = 1) due to a known family risk. Eighty percent of neonates screened positive were further investigated in seven specialised metabolic units versus 20% in local Paediatric departments.

### Process evaluation

377 data sets could be analysed for process times and process durations (Table 1). The screening process was analysed in five sequential steps from blood sampling (step 1), report of first screening result (step 2), start of confirmatory testing (step 3), confirmation of result (step 4), and start of treatment (step 5). Process times were calculated as the child's age at a particular step (days/hours for steps 1 and 2; days for steps 3 to 5) and 'process duration' as the time difference between steps. 'Start of confirmation' was defined as the start of specific investigations, except for mild hyperphenylalaninaemia, for which start of confirmation was defined as the time of the first repeat specimen.

### Outcome evaluation

The target sample for outcome evaluation included 257 cases in group 1 (excluding 88 babies with mild hyperphenylalaninaemia, six babies of mothers with maternal 3-methylcrotonyl-CoA carboxylase deficiency, four non-definitely confirmed cases), 11 patients in group 2, and 11 patients in group 3. Ten patients were soon lost to follow-up (one carnitine palmitoyltransferase II deficiency, two medium-chain acyl-CoA dehydrogenase deficiency, three short-chain acyl-CoA dehydrogenase deficiency, one biotinidase deficiency, one galactosaemia, two phenylketonuria), one patient with mitochondrial tri-functional protein deficiency deceased at the age of six months and one with non-ketotic hyperglycinaemia in the neonatal period, four were too young for outcome evaluation (≤1 year) and parents of 16 newborns did not give consent. Therefore 247 patients were eligible for outcome evaluation. Following a standardised protocol, paediatric metabolic specialists and psychologists evaluated the clinical outcome by the number of metabolic decompensations, dysfunction of selected organs, growth disturbances, standardised IQ tests (1.5 yrs Denver test, 3.5 yrs K-ABC or HAWIVA-III, 5.5 yrs HAWIK-IV) as well as school placement. Metabolic decompensation was defined as any event resulting in hospitalization after a patient showed biochemical markers of metabolic 'derangement' or clinical signs of deterioration.

## Data management and statistics

Screening data were taken from the database of the NBS centre. Confirmatory diagnostics and outcome data were retrieved from patients' files. All data were entered in standardised forms by the authors (GG, ML, PB, UW), transferred to the study's data base by a data manager (Microsoft Access 2003), checked for consistency and correctness and analyzed with SPSS Version 16.

## Results

### Part 1: Process analysis

Data sets including all five process steps were available for 205 confirmed cases. Median ages (interquartile ranges) were 2.9 days (2.34-4.00) for blood sampling (step 1), 7.1 days (5.7-9.2) for the first report of the screening result (step 2), 9.0 days (7.0-12.0) for start of confirmatory testing (step 3), 11.0 days (8.0-15.0) for confirmation of result (step 4), and 10.0 days (7.0-13.0) for start of treatment (step 5). 75% of all screening processes from steps 1 to 5 were completed within the first 13 days of life. Process times for mild hyperphenylalaninaemia vs. other cases revealed similar times for step 1 (χ^2^(1, N = 345) = 1.55; p = 0.213), but longer times for step 2 (χ^2^(1, N = 338) = 7.49; p < 0.01), step 3 (χ^2^(1, N = 336) = 13.05; p < 0.001), and step 4 (χ^2^(1, N = 334) = 44.45; p < 0.001) reflecting that for mild hyperphenylalaninaemia requests for repeat specimen were sent out by mail/fax and not communicated by phone. Median duration between steps 1 and 2 in patients who were screened according to the official German regulation (after 2005) between 36 and 72 hrs was 3.32 days.

Bonferroni adjusted Kruskal-Wallis 2-tailed tests of differences between group 1 (NBS), group 2 (symptomatic), and group 3 (high risk) were significant for age at start of NBS or specific metabolic analyses (χ^2^(2, N = 236) = 38.75; p < 0.001), age at confirmed result (χ^2^(2, N = 234) = 25.77; p < 0.001), and age at start of treatment (χ^2^(2, N = 230) = 30.11; p < 0.001) with all process times being later for group 2 than for group 3, but still earlier than for group 1. Disease specific treatment (in group 1) or supportive treatment (in group 2) often started before confirmation of diagnosis. Only in two symptomatic patients (one medium-chain acyl-CoA dehydrogenase deficiency, one galactosaemia) NBS was faster than the results of specific diagnostic investigations (Table 2). In phenylketonuria immediate start of treatment is not necessary and would even interfere with confirmatory testing. Treatment in phenylketonuria patients started less often before confirmation of diagnosis (27%) than in disorders at risk for acute metabolic decompensation (42%) (Fisher's exact test p = 0.03).

**Table 2:** Process times for patients who became symptomatic before first blood sampling or before the first screening result was available

Diagnosis	Age at decom-pensation (days)	Age at blood samp-ling 1st screening card (days)	Age at 1^st ^screening result (days)	Age at con-firmed diagnosis (days)	Age at start of treatment (days)
MCADD	0.0	4.0	11.0	12.0	14.0

MCADD	3	1.7	**6.8**	6.0	3.0

CIT I classic	1.0	3.0	**12.0**	2.0	2.0

CIT I classic	1.0	3.0	**10.0**	5.0	Not available

CIT I classic	2.0	2.7	**8.6**	2.0	2.0

IVA classic	3.0	4.4	**10.7**	8.0	8.0

PA	3.0	3.0	**16.0**	3.0	3.0

Cobalamin C/D defect	3.0	5.0	**15.0**	5.0	5.0

Galactosaemia	4.0	3.5	7.0	8.0	7.0

Galactosaemia	8.0	4.2	**11.1**	9.0	9.0

MSUD	4.0	3.4	**9.3**	5.0	5.0

Abbreviations see Table 1

Bold text indicates that the first screening result arrived after confirmed diagnosis.

### Part 2: Outcome analysis

#### Metabolic decompensations

Disorders were classified according to their risk of developing decompensation or not (see Table 1). Information on metabolic decompensation was available for 133 patients with a potentially decompensating disorder. At least one metabolic decompensation was reported for 34 (25.6%) patients: 19 out of 113 (16.8%) patients in group 1 (NBS) (see Table 1) suffered one or more decompensations, all 11 patients (100%) in group 2 (symptomatic) had altogether 28 decompensations and four out of nine patients (44%) in group 3 (high risk) had altogether 17 decompensations. All patients with classical urea cycle disorders (5/5 patients; four citrullinaemia type I, one argininosuccinate lyase deficiency) experienced at least one metabolic crisis, followed by amino acid disorders (5/10 patients; decompensations only in maple syrup urine disease), galactosaemia (6/13 patients), organic acid disorders (8/20 patients: three isovaleric aciduria, two propionic aciduria, one cobalamin C/D defect, one 3-hydroxy-3-methylglutaryl-CoA lyase deficiency, one glutaric aciduria type I), and fatty acid oxidation disorders (10/85 patients: six medium-chain acyl-CoA dehydrogenase deficiency, three long-chain 3-hydroxy-acyl-CoA dehydrogenase deficiency, one very long-chain acyl-CoA dehydrogenase deficiency). The highest number of decompensations per individual patient was observed in patients with classical citrullinaemia (one patient with six decompensations), propionic aciduria (one patient with seven decompensations) and argininosuccinate lyase deficiency (one patient with ten decompensations).

**Figure 1:**
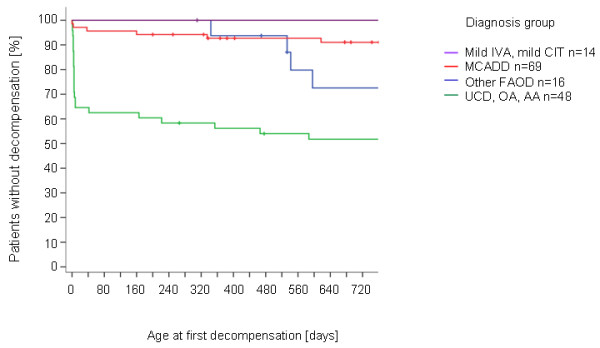
**Kaplan-Meier analysis of decompensations in disjunct subsamples of (1) mild isovaleric aciduria and mild citrullinaemia (Mild IVA, mild CIT), (2) medium-chain acyl-CoA dehydrogenase deficiency (MCADD), (3) other fatty acid oxidation disorders (FAOD), and (4) urea cycle, organic acid and amino acid disorders (UCD, OA, AA)**.

Comparison of four groups of disorders (1) mild citrullinaemia (n = 4) and mild isovaleric aciduria (n = 10), (2) medium-chain acyl-CoA dehydrogenase deficiency (n = 69), (3) fatty acid oxidation disorders other than medium-chain acyl-CoA dehydrogenase deficiency (six very long-chain acyl-CoA dehydrogenase deficiency, four long-chain acyl-CoA dehydrogenase deficiency, two carnitine transporter deficiency, three multiple acyl-CoA dehydrogenase deficiency, one carnitine palmitoyltransferase I deficiency), and (4) urea cycle disorders (five citrullinaemia type I classic, one argininosuccinate lyase deficiency), organic acid disorders (six glutaric aciduria type I, five isovaleric aciduria classic, four propionic aciduria, four methylmalonic acidurias, one 3-hydroxy-3-methylglutaryl-CoA lyase deficiency), and amino acid disorders (seven maple syrup urine disease, two tyrosinaemia type I, one 6-pyruvoyltetrahydropterin synthase deficiency) regarding their patterns of metabolic decompensation revealed a clear cut order of severity (0%, 9%, 25%, 50% decompensations, Figure 1). Kaplan-Meier analysis Mantel-Cox Log Rank test was significant for comparison between groups (1) vs. (4) (χ^2^= 9.6; p = 0.002), (1) vs. (3) (χ^2^= 4.0; p <0.05), and (4) vs. (2) (χ^2^= 26.4; p <0.0001), showed trends for (2) vs. (3) (χ^2^= 3.0; p = 0.083) and (3) vs. (4) (χ^2^= 3.5; p = 0.061). The difference between (1) and (2) was not significant (χ^2^= 1.3; p = 0.260).

#### Clinical signs, cognitive outcome and school placement

Standardised clinical status examination investigated 32 clinically relevant signs related to central nervous system, peripheral nervous system, muscle, heart, eye, liver, skin, kidney, haematopoesis and growth. For each disorder critical subsets of signs were defined (Additional file 1). Patients with ≥ 1 sign received a positive clinical status score. For 75 patients with decompensation disorders and 50 patients with phenylketonuria (total 125) at least one status evaluation was available. Out of the 75 patients with decompensation disorders, 19 (25.3%) were scored positive. Table 3 shows the highest rate of positive clinical status in symptomatically diagnosed patients (50%), while phenylketonuria and medium-chain acyl-CoA dehydrogenase deficiency had similarly low scores. Nine of 48 (18.7%) patients with a decompensation disorder but without decompensation had ≥ 1 sign compared with ten of 27 (37%) patients with at least one metabolic decompensation (OR: 2.55; 95% CI [0.88; 7.40]).

**Table 3:** Clinical status score and cognitive outcome at the age of 3 yrs, 3 months

Subsample	Positive clinical status score*; number (%)	Cognitive outcome IQ <85; number (%)
Symptomatic group	3/6 (50)	4/6 (66.7)

High risk group	1/7 (14.3)	3/7 (42.9)

NBS without MCADD and PKU	12/30 (40.0)	2/26 (7.7)

NBS: MCADD	3/32 (9.4)	1/32 (3.1)

NBS: PKU	7/50 (14.0)	4/49 (8.2)

Total	26/125 (20.8)	14/120 (11.7)

Disorders of screening panel 2005 without PKU and BIOD^$^	8/61 (13.1)	5/60 (8.3)

Additional disorders screened until 2005^#^	11/14 (78.6)	5/11 (45.5)


Abbreviations see Table 1

* ≥ 1 disorder specific clinical sign

^$ ^MSUD, Galactosaemia, MCADD, LCHADD, VLCADD, CPT ID, CPT IID, GA I, IVA

^# ^Cobalamin C/D defect, PA, HMG-CoA LD, CIT I, ASLD, TYR I, CTD, MADD

Standardised psychological assessment was done in 120 patients at a mean age of 3.3 years (SD = 1.9). Psychometric results were scored as subnormal for IQ results < 85. In the group of 70 patients with a decompensating disorder, 4 out of 44 (9.1%) without decompensation had a subnormal outcome compared with 6 out of 26 (23.1%) with at least one decompensation (OR: 3.00; 95% CI [0.76; 11.86]).

School placement is only known for 24 of 28 patients equal or older than 6 years (the target age for formal schooling in Germany). All patients with medium-chain acyl-CoA dehydrogenase deficiency (9/9) or with phenylketonuria (7/7) attend normal schools. For the more severe disorders 3/8 are not able to attend normal schools.

In the group of patients with medium-chain acyl-CoA dehydrogenase deficiency genotype was known for 28 patients. Of these 16 were homozygous for the common mutation c.985A >G (K329E). Metabolic decompensations were observed in 6 patients. In five of these, the results of neurological status and IQ tests were normal on follow up. One patient showed normal intellectual and physical development, but slight myocloni on neurological examination. The only patient in our cohort with medium-chain acyl-CoA dehydrogenase deficiency who showed severe neurological and intellectual impairment (IQ 74) never experienced a metabolic decompensation. However, he presented with severe neonatal onset cardiomyopathy, which seems to be part of a syndromatic condition and unrelated to medium-chain acyl-CoA dehydrogenase deficiency.

Comparison of disorders of the screening panel 2005 without phenylketonuria as well as biotinindase deficiency versus disorders additionally screened until 2005 showed significant differences for clinical status (OR: 24,3; 95% CI [5.5; 106]), as well as for IQ results (OR: 9.2; 95% CI [2;41]). Comparison of disorders of the screening panel 2005 without phenylketonuria and biotinidase deficiency versus phenylketonuria alone did not reveal any significant differences, neither for clinical status scores (OR: 0.9; 95% CI [0.3; 2.8]) nor for IQ results (OR: 1.0; 95% CI [0.3; 4.0]). Standardised clinical status was significantly associated with psychometric results in decompensation disorders (C = 0.44; p <0.001) as well as in phenylketonuria patients (C = 0.46; p <0.01).

## Discussion

1,084,195 newborns screened in our centre correspond to about 1.6 times the annual birth rate in Germany. As far as we know this is the first prospective single centre evaluation of a NBS programme utilizing MS/MS. Numerous publications describe the epidemiology, technical aspects and clinical validity of MS/MS screening while there are only a few retrospective evaluations of NBS programmes. Only the Australian screening programme provides data on similar aspects of overall test performance for groups of disorders as well as of follow-up results [13].

In our cohort 75% of all patients started treatment within the first 13 days of life. Out of 133 patients at risk for episodes of decompensation 11 (8%) presented clinically before the screening result was available. Even taking blood samples at 24 hrs after birth and optimal further processing of specimens would not have prevented most of these patients from early adverse events (Table 2). Kaplan-Meier-analysis revealed disorder related patterns of early and late decompensations (Figure 1). Urea cycle disorders, organic acid disorders, and amino acid disorders show the highest, earliest and continuous risk. Patients with medium-chain acyl-CoA dehydrogenase deficiency have a continuous but much lower risk for episodes of decompensation, and other fatty acid oxidation disorders an intermediate risk starting towards the end of the first year (with first intercurrent illness and/or missing feeds).

In medium-chain acyl-CoA dehydrogenase deficiency NBS leads to prevention of metabolic decompensations and neurological harm in nearly all patients [14,15], compared to 40 to 74% presenting with severe illness, 16-26% with early death and 20% developing severe neurological impairment in unscreened populations [16-18]. This benefit remains relevant although the number of MCADD cases detected is almost doubled by NBS. However, contemporary patients from unscreened cohorts surviving metabolic decompensations also showed normal neurological outcome, most probably due to improved awareness and emergency treatment [13].

Our data correspond well to those of the Australian study [13] for the common set of disorders as well as for medium-chain acyl-CoA dehydrogenase deficiency alone, except for the prevalence of symptomatic cases presenting during the first days of life (Table 4). One patient showed normal intellectual and physical development, but slight myocloni on neurological examination. As the patient's mother showed similar symptoms, these are most probably unrelated to medium-chain acyl-CoA dehydrogenase deficiency. In his brother, also with medium-chain acyl-CoA dehydrogenase deficiency, mild muscular hypotonia without any practical consequences in everyday life was observed during the standardized examination. For comparison with the results of the Australian study both ratings were judged as "nil significant". In the present study 11 out of 1,084,195 children presented clinically before the screening result was available compared to 12 of 461,500 in the Australian screened cohort (OR: 0.39; 95% CI [0.17; 0.88]). Both studies document the value of extended newborn screening mainly on the basis of medium-chain acyl-CoA dehydrogenase deficiency, a disease of European origin.

**Table 4:** Comparison of data across studies

	**Wilcken et al., 2009**[13] Screened cohort born 1998-2002 N = 461,500	This study Screened cohort born 1999-2009 N = 1,084,195	OR; 95% CI
**Prevalence group 1:**MSUD, BIOD, GA I, IVA, MCADD, CACTD, CPT ID, CPT IID; LCHADD, VLCADD	n = 37 8.0:100,000	n = 122 11.25: 100,000	1.40; [0.97;2.02]

**Prevalence group 2:**Group 1 without MCADD	n = 13 2.8: 100,000	n = 41 3.8: 100,000	1.34; [0.72;2.51]

**MCADD**			

Prevalence	n = 24 5.2: 100,000	n = 81 7.5: 100,000	1.44; [0.91;2.27]

Clinical presentation by day 5	N = 2	N = 2	

Asymptomatic or clinical presentation after day 5	N = 22	N = 79	

Metabolic decompensation during follow-up	Not reported	6/69	

Physical score nil significant at 6 (Australia)/3 (Heidelberg) years	22/22	31*/32	

No intellectual handicap at 6 (Australia)/3 (Heidelberg) years (IQ or intellectual development in normal range (IQ≥85))	22/22	31*/32	

Normal school at ~6 yrs	15/15	9/9	

Abbreviations see Table 1

* one patient never showing a metabolic decompensation but congenital cardiomyopathy of unknown origin. The clinical signs of two of three MCADD patients with a "positive" clinical status score (Table 3) were judged as non significant (see text).

Using phenylketonuria as a gold standard, patients with metabolic disorders included in the German 2005 screening panel achieved similarly good outcome data, whereas the outcome of the set of disorders screened additionally until 2005 was significantly worse than the outcome of the 2005 screening panel. Not including these disorders as a group into the screening panel could be defended as rational, although numbers are too small to draw conclusions for single disorders.

Evaluation of diseases with much lower frequencies can benefit from national and international collaboration, as could be shown for glutaric aciduria type I [19,20], as well as from comparison with historical controls, well designed "n-of-1" trials and translational research [21]. Systematic follow-up is also necessary to solve the question of mild phenotypes probably representing non-diseases.

Although unnecessary treatment of mild phenotypes of metabolic disorders is a serious problem [22], it seems unjustified to attribute the issue exclusively to NBS. Considering screening as a programme there are multiple steps to identify mild variants and revise treatment decisions. Sampling time and cut-offs influence detection rates of mild variants and the same is true for methods and cut-offs of confirmatory procedures. Duarte galactosaemia needs no further investigation and no reporting [23], but unfortunately this is not yet known for most other disorders. Therefore evaluation of the whole process including follow-up is necessary. Earlier sampling may allow earlier detection of some disorders e.g. maple syrup urine disease, but also increases the risk of missing others e.g. homocystinuria.

The principle that population screening requires a structured evaluation has been recently set in place by the US Health and Human Services Secretary's Advisory Committee on Heritable Disorders in Newborns and Children (SACHDNC) instituting a permanent review panel in 2007 [24]. Five criteria have been defined to add a condition to the NBS panel: sufficient information about the condition itself, evidence regarding appropriate screening tests, diagnostic methods, treatment and economic evaluations. In the European Union an evaluation process was recently initiated with the tender No. EAHC/2009/Health/09 'Evaluation of population newborn screening practices for rare disorders in Member States of the European Union' [25]. Reports on NBS programmes from different parts of the world are necessary to allow a comprehensive assessment of benefits, harms and costs of NBS programs [26]. As prevalences are likely to be different in populations of diverse ethnical background, pilot projects in individual countries will contribute important information [27,28]. In the Arabic Gulf country Qatar the overall frequency of metabolic disorders detected by the particular NBS program is much higher (1:966) compared to the present study (1:2920), and prevalence in a Turkish pilot study was 1:839 [29] illustrating a presumably likely high benefit of extended NBS in Turkey, Middle East and North African countries. In contrast the first comprehensive report from an East Asian country, Taiwan, revealed a prevalence of 1:6200 for all metabolic disorders, with an exceedingly low yield of fatty acid oxidation disorders, one of the main justifications for MS/MS screening in Caucasian populations [30].

We have presented the data of a single centre longitudinal registry so that they can be compared with others. Aside from economic evaluation all the criteria set by the SACHDNC [24] for extended NBS were addressed. We could demonstrate that physical and cognitive outcome of patients with presymptomatic diagnosis of metabolic disorders included in the current German screening panel is equally good as in patients with phenylketonuria. However, the specific evaluation of most of the rare disorders is still necessary and will require international registries and collaborative studies.

## Abbreviations

NBS**: **newborn screening; MS/MS: tandem-mass spectrometry

## Competing interests

The authors declare that they have no competing interests.

## Authors' contributions

All authors were involved in collection of data, critical revision and final approval of the article: GFH and ML conceived the idea. GFH is guarantor. PB and GH performed the statistical analysis. GG monitored data collection. ML and PB wrote the initial draft of the manuscript and all subsequent drafts with input from all co-authors.

All authors had full access to all of the data and can take responsibility for the integrity of the data and the accuracy of the data analysis.

The sponsor of the study had no role in the study design, data collection, data analysis, data interpretation, writing of the report, or the decision to submit for publication.

## Ethical approval

The study was approved by the IRB of the Medical Faculty of the University Hospital Heidelberg (IRB code 104/2005) and parents of all participants gave written informed consent.

## Funding

This extensive study over more than a decade was only made possible by the continuous and generous support of the Dietmar Hopp Foundation, St. Leon-Rot, Germany.

## Note

* Dedicated to Dietmar Hopp on the occasion of his 70th birthday

## Supplementary Material

Additional file 1 Supplementary Table S1 Click here for file
