# Supplementary material for: Efficacy and outcome of expanded newborn screening for metabolic diseases - Report of 10 years from South-West Germany *
Source: Orphanet J Rare Dis. 2011 Jun 20;6:44. doi: 10.1186/1750-1172-6-44 (PMC3141366; doi:10.1186/1750-1172-6-44)
Supplement: Additional file 1 — Supplementary Table S1 [file 1750-1172-6-44-S1.DOC]

**Supplementary Table S1. Subsets of critical signs in clinical status evaluation per disorder**

| **Disorder** | **Psychomotor retardation** | **Psychomotor regression** | **Episodes of coma/ somnolence/ hypoglycemia** | **Muscular hypotonia** | **Muscular hypertonia** | **Pyramidal signs** | **Extrapyramidal signs** | **Cerebellar signs** | **Brain stem signs** | **Myoclonus** | **Epilepsy** | **Neuropathy** | **Rhabdomyolysis** | **Exercise Intolerance** | **Weakness** | **Myalgia** | **Cardiomyopathy** | **Retinal pigment degeneration** | **Optic atrophy** | **Cataract** | **Scotoma** | **Acute liver failure** | **Chronic liver failure** | **Elevated serum transaminases** | **Skin rash/Eczema** | **Fanconi syndrome** | **Other tubulopathies** | **Renal failure** | **Pancytopaenia** | **Neutropaenia** | **Weight < p3 or crossing percentiles** | **Length/Height < p3 or crossing percentiles** | **Number of relevant clinical signs/ disorder** |
| --- | --- | --- | --- | --- | --- | --- | --- | --- | --- | --- | --- | --- | --- | --- | --- | --- | --- | --- | --- | --- | --- | --- | --- | --- | --- | --- | --- | --- | --- | --- | --- | --- | --- |
| **3-MCCD** | 1 | 1 | 1 | 1 | 1 | 1 | 1 |  |  |  |  |  |  | 1 | 1 |  |  |  |  |  |  | 1 |  |  | 1 |  |  |  |  | 1 | 1 | 1 | 14 |
| **ASLD** | 1 | 1 | 1 | 1 | 1 | 1 | 1 | 1 | 1 | 1 | 1 |  |  |  | 1 |  |  |  |  |  |  | 1 | 1 | 1 |  |  |  |  |  |  | 1 | 1 | 17 |
| **BIOD** | 1 | 1 | 1 | 1 | 1 | 1 | 1 | 1 | 1 | 1 | 1 | 1 | 1 | 1 | 1 |  |  |  |  |  |  | 1 | 1 | 1 | 1 |  |  |  |  |  | 1 | 1 | 21 |
| **CTD** | 1 | 1 | 1 | 1 | 1 | 1 | 1 | 1 | 1 | 1 | 1 |  | 1 | 1 | 1 | 1 | 1 |  |  |  |  |  |  | 1 |  |  |  |  |  |  | 1 | 1 | 19 |
| **MMA/Cbl** | 1 | 1 | 1 | 1 | 1 | 1 | 1 | 1 | 1 | 1 | 1 | 1 |  | 1 | 1 |  |  |  | 1 |  | 1 |  |  |  |  |  |  |  |  |  | 1 | 1 | 18 |
| **CIT I** | 1 | 1 | 1 | 1 | 1 | 1 | 1 | 1 | 1 | 1 | 1 |  |  |  |  |  |  |  |  |  |  |  |  |  |  |  |  |  |  |  | 1 | 1 | 13 |
| **CPT ID** | 1 | 1 | 1 | 1 | 1 | 1 | 1 | 1 | 1 | 1 | 1 |  |  |  |  |  |  |  |  |  |  |  |  |  |  |  |  |  |  |  | 1 | 1 | 13 |
| **CPT IID** | 1 | 1 | 1 | 1 | 1 | 1 | 1 | 1 | 1 | 1 | 1 |  | 1 | 1 | 1 | 1 | 1 |  |  |  |  |  |  |  |  | 1 | 1 | 1 |  |  | 1 | 1 | 21 |
| **GA I** | 1 | 1 | 1 | 1 | 1 | 1 | 1 | 1 | 1 | 1 | 1 |  |  |  |  |  |  |  |  |  |  |  |  |  |  |  |  |  |  |  | 1 | 1 | 13 |
| **Galactosaemia** | 1 | 1 |  |  |  |  |  |  |  |  |  |  |  |  |  |  |  |  |  | 1 |  | 1 | 1 |  |  |  |  |  |  |  | 1 | 1 | 7 |
| **CACTD** | 1 | 1 | 1 | 1 | 1 | 1 | 1 | 1 | 1 | 1 | 1 |  | 1 | 1 | 1 | 1 | 1 |  |  |  |  |  |  |  |  | 1 | 1 |  |  |  | 1 | 1 | 20 |
| **HMG-CoA LD** | 1 | 1 | 1 | 1 | 1 | 1 | 1 | 1 | 1 | 1 | 1 | 1 |  |  |  |  |  |  |  |  |  |  |  | 1 |  |  |  |  |  |  | 1 | 1 | 15 |
| **IVA** | 1 | 1 | 1 | 1 | 1 | 1 | 1 | 1 | 1 | 1 | 1 |  |  |  |  |  |  |  |  |  |  |  |  |  |  |  |  |  | 1 | 1 | 1 | 1 | 15 |
| **LCHADD/mTFP** | 1 | 1 | 1 | 1 | 1 | 1 | 1 | 1 | 1 | 1 | 1 | 1 | 1 | 1 | 1 | 1 | 1 | 1 | 1 |  | 1 | 1 |  | 1 |  |  |  |  |  |  | 1 | 1 | 24 |
| **MADD** | 1 | 1 | 1 | 1 | 1 | 1 | 1 | 1 | 1 | 1 | 1 | 1 | 1 | 1 | 1 | 1 | 1 | 1 | 1 |  |  | 1 | 1 | 1 |  |  |  | 1 |  |  | 1 | 1 | 25 |
| **MCADD** | 1 | 1 | 1 | 1 | 1 | 1 | 1 | 1 | 1 | 1 | 1 |  |  |  |  |  |  |  |  |  |  |  |  |  |  |  |  |  |  |  | 1 | 1 | 13 |
| **MSUD** | 1 | 1 | 1 | 1 | 1 | 1 | 1 | 1 | 1 | 1 | 1 |  |  |  |  |  |  |  |  |  |  |  |  |  |  |  |  |  |  |  | 1 | 1 | 13 |
| **PKU*** | 1 | 1 |  |  | 1 | 1 |  |  |  | 1 | 1 |  |  |  |  |  |  |  |  |  |  |  |  |  | 1 |  |  |  |  |  | 1 | 1 | 9 |
| **PA** | 1 | 1 | 1 | 1 | 1 | 1 | 1 |  |  |  | 1 |  |  | 1 | 1 | 1 | 1 |  |  |  |  |  |  | 1 | 1 |  |  |  | 1 | 1 | 1 | 1 | 18 |
| **SCADD** |  |  |  |  |  |  |  |  |  |  |  |  |  |  |  |  |  |  |  |  |  |  |  |  |  |  |  |  |  |  |  |  | 0 |
| **TYR I** | 1 | 1 | 1 | 1 | 1 | 1 |  |  |  |  |  | 1 |  | 1 | 1 |  |  | 1 | 1 | 1 | 1 | 1 | 1 |  |  | 1 | 1 | 1 |  |  | 1 | 1 | 20 |
| **TYR III** |  |  |  |  |  |  |  | 1 |  |  | 1 |  |  |  |  |  |  |  |  |  |  |  |  |  |  |  |  |  |  |  | 1 | 1 | 4 |
| **VLCADD** | 1 | 1 | 1 | 1 | 1 | 1 | 1 | 1 | 1 | 1 | 1 |  | 1 | 1 | 1 | 1 | 1 |  |  |  |  | 1 |  |  |  |  |  |  |  |  | 1 | 1 | 19 |
|  | **1** | **2** | **3** | **4** | **5** | **6** | **7** | **8** | **9** | **10** | **11** | **12** | **13** | **14** | **15** | **16** | **17** | **18** | **19** | **20** | **21** | **22** | **23** | **24** | **25** | **26** | **27** | **28** | **29** | **30** | **31** | **32** |  |

* including one patient with PTPSD

1 = relevant clinical sign for this disorder

Abbreviations: ASLD = Argininosuccinate lyase deficiency; BIOD = Biotinidase deficiency; CACTD = Carnitine acylcarnitine translocase deficiency; CIT I = Citrullinemia type I; CPT ID = Carnitine palmitoyltransferase I deficiency; CPT IID = Carnitine palmitoyltransferase II deficiency; CTD = Carnitine transporter deficiency; GA I = Glutaric aciduria type I; HMG-CoA LD = 3-Hydroxy-3-methylglutaryl-CoA lyase deficiency; IVA = Isovaleric aciduria; LCHADD = Long-chain 3-hydroxy-acyl-CoA dehydrogenase deficiency; mTFP = mitochondrial tri-functional protein deficiency; MADD = Multiple acyl-CoA dehydrogenase deficiency; MCADD = Medium-chain acyl-CoA dehydrogenase deficiency; 3-MCCD = 3-Methylcrotonyl-CoA carboxylase deficiency; MMA/Cbl = Methylmalonic acidurias (all kinds); MSUD = Maple syrup urine disease; PA = Propionic aciduria; PKU = Phenylketonuria; PTPSD = 6-Pyruvoyltetrahydropterin synthase deficiency; SCADD = Short-chain acyl-CoA dehydrogenase deficiency; TYR I/III = Tyrosinaemia type I/III; VLCADD = Very long-chain acyl-CoA dehydrogenase deficiency
